# Dechlorination of mixed chlorinated organophosphate esters (V6 and TCEP) and associated reductive dehalogenase gene expression by *Dehalococcoides mccartyi*

**DOI:** 10.1128/aem.01990-25

**Published:** 2026-01-06

**Authors:** Sen Yang, Junhong Wu, Qian Yang, Yirong Deng, Heli Wang, Dan Li, Lihua Yang, Jianzhong Song, Yin Zhong, Ping'an Peng

**Affiliations:** 1 State Key Laboratory of Advanced Environmental Technology, Guangzhou Institute of Geochemistry, Chinese Academy of Sciences 540073 , Guangzhou, China; 2 Guangdong Key Laboratory of Environmental Protection and Resources and Utilization, Guangzhou, China; 3 Guangdong-Hong Kong-Maco Joint Laboratory for Environmental Pollution and Control, Guangzhou, China; 4 University of Chinese Academy of Sciences 74519, Beijing, China; 5 Guangdong Key Laboratory of Contaminated Sites Environmental Management and Remediation, Guangdong Provincial Academy of Environmental Science 605149, Guangzhou, China; 6 School of Environment and Civil Engineering, Dongguan University of Technology 74549, Dongguan, China; 7 South China Sea Resource Exploitation and Protection Collaborative Innovation Center, Guangdong Provincial Key Laboratory of Marine Resources and Coastal Engineering, School of Marine Sciences, Sun Yat-Sen University 26469 , Guangzhou, China; Shanghai Jiao Tong University, Shanghai, China

**Keywords:** chlorinated organophosphate esters, commercial V6, TCEP, *Dehalococcoides mccartyi*, reductive dehalogenase

## Abstract

*Dehalococcoides mccartyi* (*D. mccartyi*) plays a critical role in the dechlorination of halogenated organic pollutants, yet its performance on mixed chlorinated organophosphate esters (Cl-OPEs) remains poorly understood. In this study, a mixed microbial culture containing *D. mccartyi* was developed to dechlorinate commercial V6, a mixture comprising 80.2% 2,2-bis(chloromethyl)trimethylene bis(bis(2-chloroethyl) phosphate) (V6), 9.4% tris(2-chloroethyl) phosphate (TCEP), and other Cl-OPE impurities. Within 10 days, 99.5% of TCEP and 95.4% of V6 were dechlorinated. The presence of TCEP slightly enhanced the dechlorination of V6 compared with V6 as a purified compound. Both compounds underwent cleavage of C−O and C−Cl bonds in their chloroethoxy groups (Cl–CH_2_–CH_2_–O–), yielding different phosphate de-esterification products and ethene. *D. mccartyi* exhibited significant growth, with two reductive dehalogenase homologous (*rdhA*) genes co-transcribed during the dechlorination of commercial V6, purified V6, or TCEP. Metaproteomic analysis revealed that the enzymes encoded by these two genes were significantly expressed, suggesting that they may be the key enzymes mediating the dechlorination of mixed Cl-OPEs. Overall, this study provides insights into the role of *D. mccartyi* and its reductive dehalogenases in the natural attenuation of mixed Cl-OPEs in contaminated environments.

**IMPORTANCE:**

Commercial V6, a chlorinated organophosphate esters mixture, is widely used in polyurethane foam and has been detected in various environmental matrices. This study is the first to elucidate the microbial transformation pathways and mechanisms of commercial V6. A mixed culture containing *Dehalococcoides mccartyi* was found to dechlorinate V6 and tris(2-chloroethyl) phosphate (TCEP) into phosphate de-esterification products, chloride ion, and ethene. Notably, two reductive dehalogenase genes were simultaneously transcribed and their corresponding enzymes co-expressed, indicating a key role of *D. mccartyi* in the natural attenuation of commercial V6 in the environment.

## INTRODUCTION

With the phase-out of certain brominated flame retardants, chlorinated organophosphate esters (Cl-OPEs) are increasingly being used as alternative flame retardants in various consumer products (1, 2). Commonly used Cl-OPEs include tris(2-chloroethyl) phosphate (TCEP), tris(1-chloro-2-propyl) phosphate (TCPP), tris(1,3-dichloro-2-propyl) phosphate (TDCPP), and 2,2-bis(chloromethyl)trimethylene bis(bis(2-chloroethyl) phosphate) (V6) (1, 3). Commercial V6 is a mixture of Cl-OPEs typically containing V6 (>80%, wt/wt), TCEP (4.5%–13.5%, wt/wt), and other Cl-OPEs impurities (4). Commercial V6 is widely used in polyurethane foam, particularly in the automotive industry, with concentrations in foam reaching up to 12% (5–7). The annual production volume of V6 reached an estimated 2,400 tons in 2020 (4, 8). However, commercial V6 exhibits potential neurotoxicity and developmental toxicity in rats, along with high persistence compared to other commonly used Cl-OPEs (3, 4). Commercial V6, as an emerging organohalide contaminant, has been of concern (4).

The widespread application of commercial V6 has resulted in the co-contamination of V6 and TCEP across various environmental matrices, including house dust, car dust, fish, water, and sediment (9–12). For example, the median concentration of V6 and TCEP in the car dust was 103 and 1,080 ng g^−1^, respectively (9). End-of-life vehicle (ELV) dismantling sites are notorious hotspots for Cl-OPEs contamination (12). Sediment samples collected inside and outside the pipelines at an ELV dismantling site in Hokksund, Norway, contained 17,559 to 30,250 mg kg^−1^ loss of ignition weight (LOI) of Cl-OPEs (13). Among these, V6 concentrations ranged from 1,200 to 2,800 mg kg^−1^ LOI, and TCEP concentrations ranged from 2,300 to 5,500 mg kg^−1^ LOI. V6 and TCEP have also been detected in over 50% of surface water samples from Wuhan, China, where the automotive and auto parts industries are a primary pillar of the local economy (14). Understanding the natural attenuation process of commercial V6 in the environment, particularly at ELV dismantling sites, is critical for evaluating its environmental persistence and assessing potential risks to ecosystems.

Organohalide-respiring bacteria are key contributors to the natural attenuation of organohalide contaminants (15). In an anaerobic culture (culture 8E) enriched from e-waste contaminated river sediments and containing *Dehalococcoides mccartyi* (*D. mccartyi*), TCEP was reductively transformed into bis(2-chloroethyl) phosphate (BCEP), ethene, and chloride ions (16). To verify the origin of ethene, a deuterium-labeled experiment was conducted. The detection of non-deuterated ethene indicated that the hydrogen atoms in the chloroethoxy group (Cl–CH_2_–CH_2_–O–) remained unchanged during ethene formation. This result ruled out both reductive dechlorination (hydrogenolysis) and dehydrochlorination mechanisms, as these would involve the addition or loss of hydrogen, thereby altering the isotopic composition of the resulting ethene. Instead, the authors proposed that TCEP reduction proceeds via cleavage of the C–Cl and C–O bonds, leading to ethene generation without hydrogen isotope exchange. A similar dechlorination pathway was observed for TCPP, which was converted to bis(1-chloro-2-propyl) phosphate and propene by another *D. mccartyi*-containing mixed culture (16). Commercial V6 shares functional groups and structural motifs with TCEP and TCPP, suggesting that *D. mccartyi* reductively transforms these compounds via similar pathways. Reductive dechlorination is typically catalyzed by reductive dehalogenase (RDase). Although a previous study has reported active transcription of reductive dehalogenase homologous genes (*rdhA*) during TCEP and TCPP dechlorination (16), the specific RDases responsible for Cl-OPEs transformation in *D. mccartyi* remain unidentified. To bridge this knowledge gap, it is essential to investigate the transcription of *rdhA* genes and corresponding RDase expression during anaerobic Cl-OPEs dechlorination, thereby clarifying the role of RDases in this process.

In this study, a mixed culture containing *D. mccartyi*, designated ZNV, was enriched from an ELV dismantling site using commercial V6 as an electron acceptor. Our objectives were to compare the dechlorination extent, products, and pathways of V6 and TCEP, both individually and as components of commercial V6, as well as to profile the expression of *rdhA* genes in culture ZNV. We monitored the growth of *D. mccartyi* during the dechlorination of different Cl-OPEs using quantitative PCR (qPCR). Metagenomic binning, reverse transcription quantitative PCR (RT-qPCR), and metaproteomic analyses were further employed to assess the transcription and expression of *rdhA* genes throughout the dechlorination process. The findings from this study will enhance our understanding of the microbial transformation processes of commercial V6 at ELV dismantling sites and clarify the role of *D. mccartyi* in the natural attenuation of mixed Cl-OPEs in contaminated environments.

## RESULTS AND DISCUSSION

### Transformation of commercial V6, purified V6, and TCEP

The commercial V6 used in this study was composed of 80.2% V6, 9.4% TCEP, and 10.4% other impurities. Among these impurities, three compounds were identified: 3-chloro-2-(chloromethyl)-2-(hydroxymethyl)propyl bis(2-chloroethyl) phosphate (abbreviated as BCEP-BCMHp; chemical formula C_9_H_17_Cl_4_O_5_P), bis(2-chloroethyl) (3-chloroprop-1-en-1-yl) phosphate (abbreviated as BCECpP; C_7_H_12_Cl_3_O_4_P), and bis(2-chloroethyl) vinylphosphonate (abbreviated as BCEvP; C_6_H_11_Cl_2_O_3_P). Detailed identification data for these compounds are provided in Table S1, Fig. S1, and Fig. S2. Due to the absence of authentic standards, the concentrations of BCEP-BCMHp, BCECpP, and BCEvP were not quantified.

**Fig 1:**
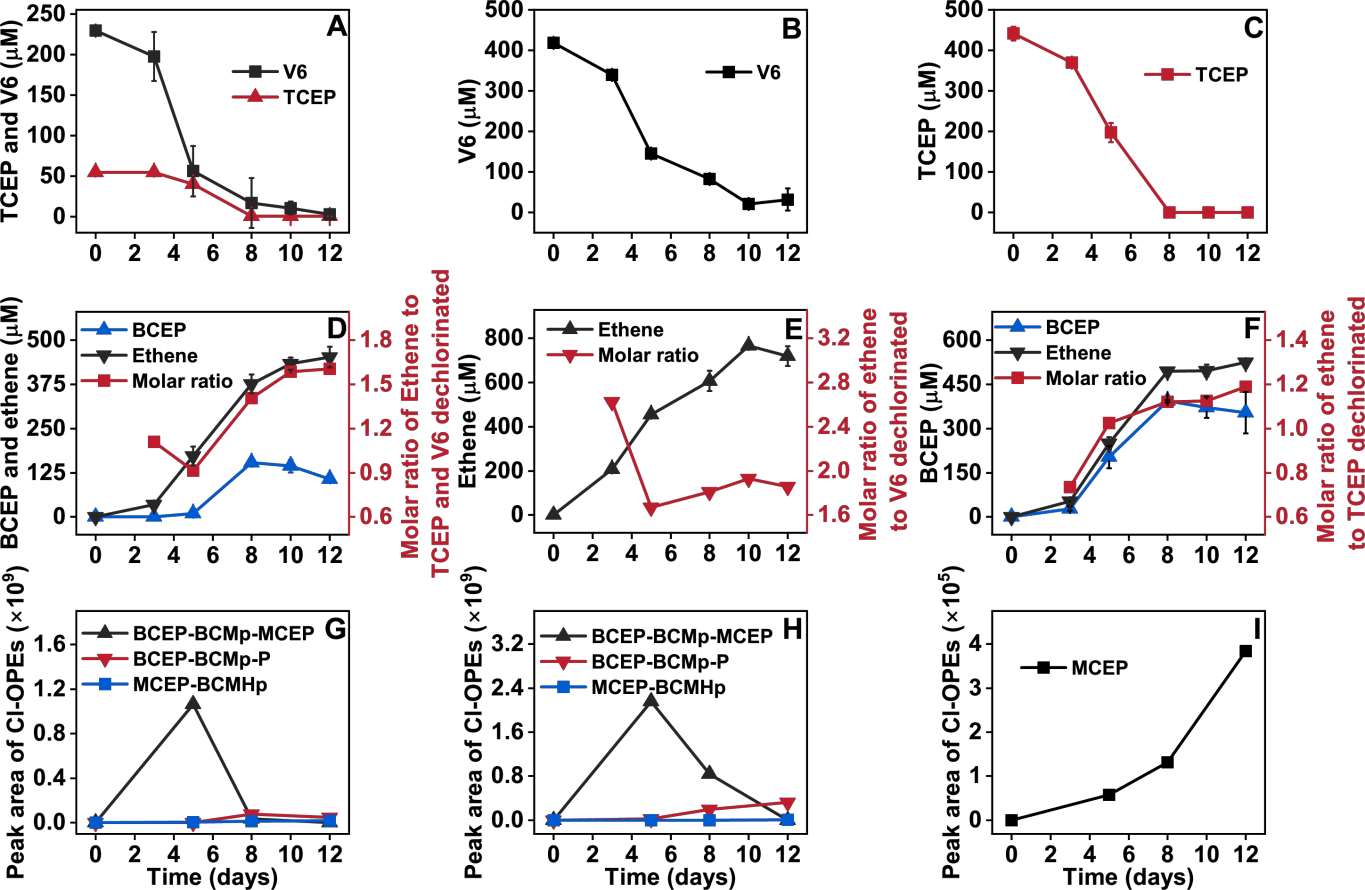
Anaerobic transformation of commercial V6 (**A, D, G**), purified V6 (**B, E, H**), and TCEP (**C, F, I**) by the culture ZNV. (**A–C**) Transformation kinetics; (**D–E**) BCEP and ethene formation, as well as the molar ratio of ethene to TCEP or/and V6 dechlorinated; (**G–I**) extracted ion chromatogram peak areas of transformation products obtained by UHPLC-Orbitrap Fusion Lumos Tribrid mass spectrometry.

Culture ZNV demonstrated efficient dechlorination of commercial V6, purified V6, and TCEP (Fig. 1A through C). Within 10 days, complete transformation (100%) of TCEP and 94.9% of purified V6 were observed. In the case of commercial V6, 99.5% of the TCEP component and 95.4% of V6 were transformed during the same period. These results indicate that ZNV exhibited higher transformation efficiency toward TCEP than for V6, regardless of whether the compounds were present individually or within the commercial mixture. Notably, during the first 8 days, V6 transformation proceeded more rapidly in the presence of TCEP (92.8%) compared to purified V6 alone (80.2%), suggesting that TCEP may facilitate the V6 transformation in the commercial formulation. Abiotic losses of Cl-OPEs were less than 3% degradation observed after 10 days in sterilized control cultures (Fig. S3).

### Dechlorination products and pathways of commercial V6, purified V6, and TCEP

**Fig 2:**
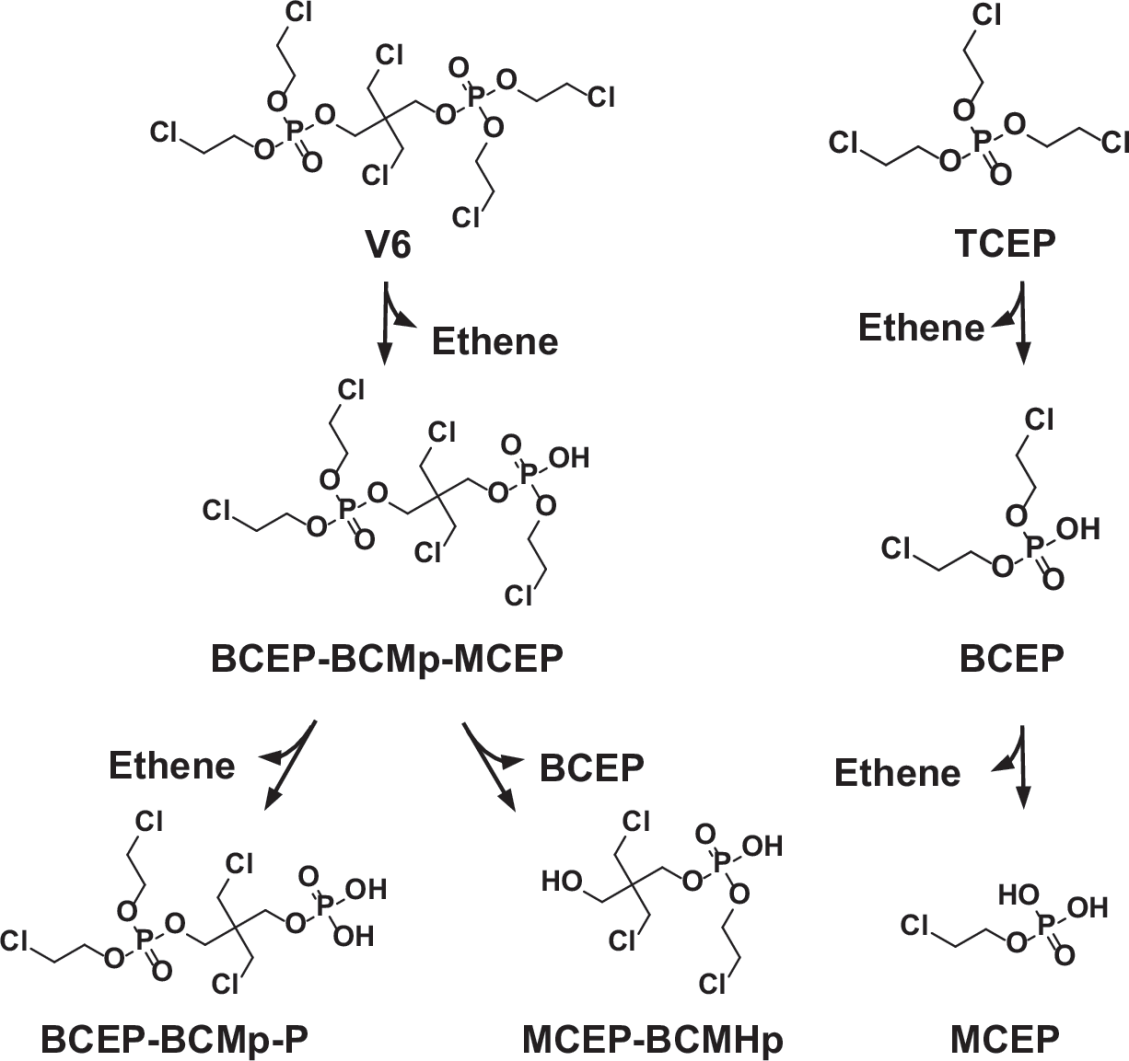
Proposed degradation pathways of V6 and TCEP by the culture ZNV using commercial V6 as electron acceptors.

During dechlorination by culture ZNV, six, five, and three intermediates were detected for commercial V6, purified V6, and TCEP, respectively (Table 1 and Fig. 2). Detailed identification data for these compounds are provided in Table S2, Fig. S1, and Fig. S2. For TCEP, the dechlorination intermediates included BCEP, mono-chloroethyl phosphate (MCEP; C_2_H_6_ClO_4_P), and ethene. These results align with a previous study on TCEP dechlorination by enrichment culture ZNE, also originating from the ELV dismantling site (17). We propose that TCEP is reduced through cleavage of the C−Cl bond and the C−O bond within the chloroethoxy group, yielding BCEP and ethene. Subsequently, BCEP undergoes a similar reductive pathway to form MCEP and ethene (Fig. 2).

**TABLE 1:** Information on V6, TCEP, and their transformation products from UHPLC-Orbitrap Fusion TMS spectra

Abbreviation	RT (min)	Formula	[M + H]^+^ (m/z)		Mass error (ppm)	RDB
			Theoretical	Experimental		
V6	10.46	C_13_H_24_Cl_6_O_8_P_2_	580.915	580.9151	0.132	−0.5
BCEP-BCMp-MCEP	9.89	C_11_H_21_Cl_5_O_8_P_2_	518.9227	518.9228	0.278	−0.5
TCEP	8.92	C_6_H_12_Cl_3_O_4_P	284.9612	284.9612	0.123	−0.5
MCEP-BCMHp	8.11	C_7_H_14_Cl_3_O_5_P	314.9717	314.9717	0.065	−0.5
BCEP-BCMp-P	7.23	C_9_H_18_Cl_4_O_8_P_2_	456.9306	456.9304	0.377	−0.5
BCEP	5.28	C_4_H_9_Cl_2_O_4_P	222.9688	222.9688	−0.032	−0.5
MCEP	2.32	C_2_H_6_ClO_4_P	160.9765	160.9765	−0.307	−0.5

For purified V6, the dechlorination intermediates included 3-((bis(2-chloroethoxy)phosphoryl)oxy)-2,2-bis(chloromethyl)propyl (2-chloroethyl) hydrogen phosphate (BCEP-BCMp-MCEP; C_11_H_21_Cl_5_O_8_P_2_), 3-((bis(2-chloroethoxy)phosphoryl)oxy)-2,2-bis(chloromethyl)propyl dihydrogen phosphate (BCEP-BCMp-P; C_9_H_18_Cl_4_O_8_P_2_), 3-chloro-2-(chloromethyl)-2-(hydroxymethyl)propyl (2-chloroethyl) hydrogen phosphate (MCEP-BCMHp; C_7_H_14_C_3_O_5_P), BCEP, and ethene. These intermediates suggest that V6 undergoes dechlorination via mechanisms similar to those observed for TCEP. Specifically, V6 may undergo cleavage of the C−Cl bond and C−O bonds in the chloroethoxy group, yielding compounds, such as BCEP-BCMp-MCEP, BCEP-BCMp-P, and ethene (Fig. 2). Furthermore, BCEP-BCMp-MCEP may be hydrolyzed to produce MCEP-BCMHp and BCEP.

During the transformation of commercial V6, the intermediates detected included BCEP-BCMp-MCEP, BCEP-BCMp-P, MCEP-BCMHp, BCEP, 2-chloroethyl (3-chloroprop-1-en-1-yl) hydrogen phosphate (CECpP; C_5_H_9_Cl_2_O_4_P), and ethene (Table 1; Table S1). Among these, BCEP-BCMp-MCEP and BCEP-BCMp-P were derived from V6, whereas BCEP originated either from TCEP or through the transformation of BCEP-BCMp-MCEP (Fig. 2). The intermediates MCEP-BCMHp and CECpP likely resulted from the dechlorination of impurities present in the commercial mixture (Fig. S4). It is proposed that BCEP-BCMHp and BCECpP undergo cleavage of the C−Cl and C−O bonds in their chloroethoxy groups, yielding MCEP-BCMHp and CECpP, respectively. Notably, MCEP was not detected during commercial V6 dechlorination, possibly due to competitive interactions among the mixed Cl-OPEs that inhibited the further reduction of BCEP to MCEP.

The production of ethene significantly increased during the dechlorination of commercial V6, purified V6, and TCEP (Fig. 1D through F). After 12 days of incubation, the accumulated ethene reached 451.9 µM from commercial V6 (initially containing 55.6 µM TCEP and 229.5 µM V6), 718.9 µM from purified V6 (initial concentration 418.4 µM), and 525.0 µM from TCEP (initial concentration 441.5 µM). Theoretically, one mole of TCEP is dechlorinated to yield one mole of BCEP and one mole of ethene. The experimental molar ratio of ethene produced to TCEP dechlorinated was slightly above 1.0, whereas the ratio of BCEP produced to TCEP consumed was below 1.0 (Fig. 1F). These results suggest that although most TCEP was converted to BCEP and ethene, a small fraction of BCEP underwent further reduction to MCEP (Fig. 1I) and additional ethene.

During the dechlorination of V6, the molar ratio of ethene produced to V6 dechlorinated reached 1.93 after 12 days of incubation (Fig. 1E), suggesting that V6 was primarily dechlorinated into BCEP-BCMp-MCEP and ethene, with the majority of BCEP-BCMp-MCEP subsequently reduced to BCEP-BCMp-P and additional ethene. This interpretation is corroborated by the lower peak area of BCEP-BCMp-MCEP relative to BCEP-BCMp-P after 12 days (Fig. 1H).

Similarly, during the dechlorination of commercial V6, the molar ratio of ethene produced to the amount of TCEP and V6 dechlorinated reached 1.60 after 12 days of incubation (Fig. 1D). Ethene generation was mainly attributed to the dechlorination of V6 into BCEP-BCMp-MCEP and BCEP-BCMp-P, with additional ethene originating from the transformation of TCEP to BCEP, BCEP-BCMHp to MCEP-BCMHp, and BCECpP to CECpP (Fig. 1G). Notably, the molar ratio of BCEP produced to TCEP transformed exceeded 2.0, suggesting that BCEP can also be derived from the dechlorination of V6 or impurities within commercial V6 (Fig. 2).

### The microbial composition of culture ZNV and *Dehalococcoides* growth

**Fig 3:**
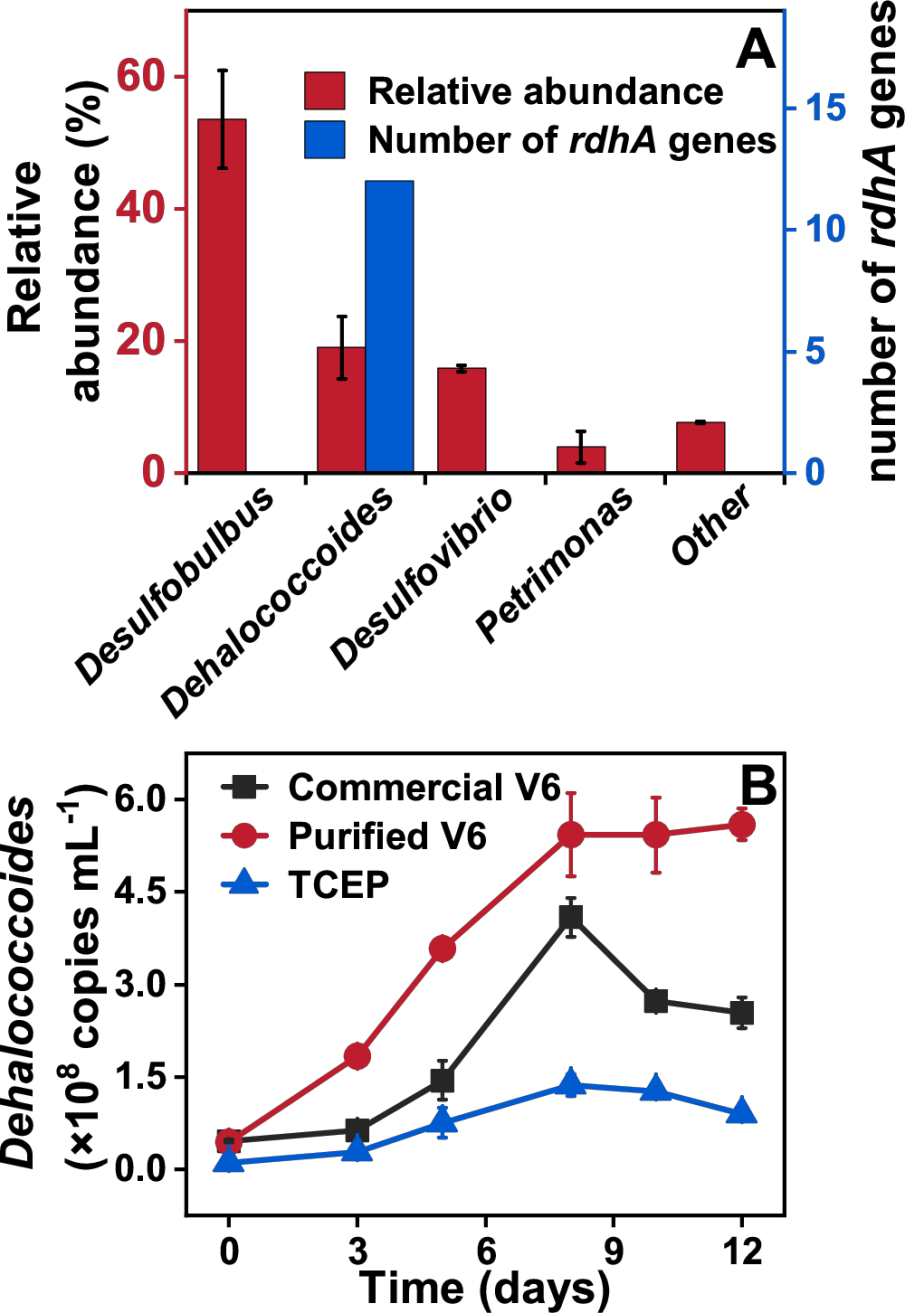
Microbial community composition (**A**) and growth of *Dehalococcoides* (**B**) in the culture ZNV fed with commercial V6 (black), purified V6 (red), and TCEP (blue), respectively. The microbial community composition with relative abundance >1%, analyzed using 16S rRNA gene amplicon sequencing, was shown.

The microbial community structure of the mixed culture ZNV was analyzed using 16S rRNA gene amplicon sequencing. The dominant genera identified were *Desulfobulbus*, *Dehalococcoides,* and *Desulfovibrio,* with relative abundances of 53.6% ± 7.4%, 19.0% ± 4.8%, and 15.8% ± 0.4%, respectively (Fig. 3A). Both *Desulfobulbus* and *Desulfovibrio* are known to utilize hydrogen as an electron donor and acetate as a carbon source. Similarly, *Dehalococcoides*, an obligate organohalide-respiring bacterium, also depends on hydrogen and acetate for growth (18–20). This metabolic overlap suggests potential competition among these genera for hydrogen and acetate.

*Dehalococcoides* has been previously shown to mediate the reductive transformation of TCEP into BCEP and ethene (16, 17). During the dechlorination of commercial V6, purified V6, and TCEP, the abundance of *Dehalococcoides* increased, reaching a peak at day 8 (Fig. 3B), indicating a pivotal role of this genus in the dechlorination of Cl-OPEs. When fed with commercial V6, purified V6, or TCEP, the cell number of *Dehalococcoides* increased from initial values of 4.6 × 10^7^, 4.4 × 10^7^, and 1.0 × 10^7^ cells mL^−1^ to 4.1 × 10^8^, 5.4 × 10^8^, and 1.4 × 10^8^ cells mL^−1^ by day 8, respectively (Fig. 3B; Fig. S5). The corresponding cell yields were calculated as 8.4 × 10^14^, 8.2 × 10^14^, and 2.9 × 10^14^ cells per mole of chlorine released, based on the stoichiometric relationship that one mole of ethene produced corresponds to one mole of chloride released.

Notably, the cell yields of *Dehalococcoides* in culture ZNV grown on commercial V6 or purified V6 were higher than those observed in other Cl-OPE-degrading cultures (such as ZNE, 8E, and 8P), which ranged from 0.25 × 10^14^ to 6.4 × 10^14^ cells per mole of chlorine released (16, 17). These yields also exceeded those reported for *Dehalococcoides* grown with chloroalkenes, halogenated benzenes, or polycyclic halogenated compounds, typically ranging from 0.2 × 10^14^ to 5.6 × 10^14^ cells per mole of chlorine released (21–25). These findings suggest that commercial V6 and purified V6 support greater growth of *Dehalococcoides* compared to TCEP, TCPP, and other organohalides, highlighting their potential as favorable substrates for *Dehalococcoides*-mediated bioremediation.

However, in culture ZNV fed with commercial V6 or TCEP, the abundance of *Dehalococcoides* declined after 8 days of incubation. This pattern is consistent with previous studies on the dechlorination of TCEP, perchloroethene, and aromatic organohalides by *Dehalococcoides*-containing cultures or pure strains (16, 17, 26–28). The decrease in *Dehalococcoides* density may be attributed to the toxic effects of accumulated BCEP during the dechlorination of commercial V6 or TCEP. In contrast, when grown on purified V6, the density of *Dehalococcoides* stabilized after reaching its peak, which can be attributed to significantly lower BCEP accumulation compared to commercial V6 and TCEP. This observation underscores the influence of intermediate toxicity on microbial dynamics during Cl-OPEs dechlorination.

### Analysis of *rdhA* gene transcription and putative RDases expression

Metagenomic analysis showed that all annotated *rdhA* genes belonged to *D. mccartyi* (Fig. 3A). Only one draft genome bin (99% completeness and <1% contamination) of *D. mccartyi*, named BinZNV2024, was successfully reconstructed from the culture ZNV. The results suggested that only one *D*. *mccartyi* sp. was present in the culture ZNV. A maximum-likelihood phylogenetic tree was constructed to compare the genomes of BinZNV2024 and 27 representative *D. mccartyi* strains (Fig. S6), which suggested that BinZNV2024 belonged to the *D. mccartyi* subgroup Cornell.

**Fig 4:**
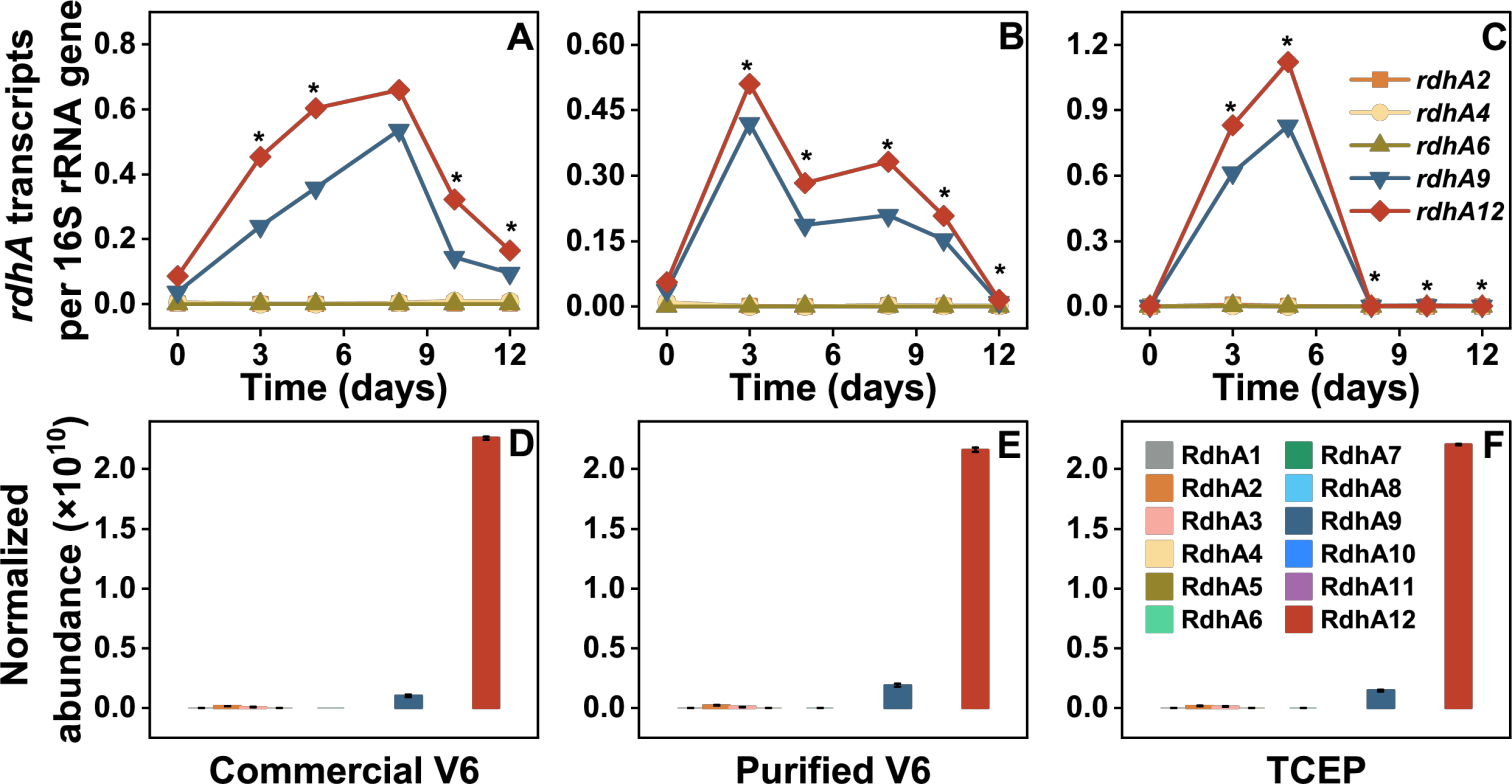
Transcription (**A–C**) of *rdhA* genes and the expression of RdhA (**D–F**) in the culture ZNV fed with commercial V6 (**A, D**), purified V6 (**B, E**), and TCEP (**C, F**), respectively. The *rdhA* genes (1, 3, 5, 7, 8, 10, and 11) displaying no transcription were not shown in the A–C. Significant differences between the transcript levels of *rdhA12* and *rdhA9* at each sampling time point are indicated by asterisks (^∗^: *P* < 0.05).

A total of 12 *rdhA* genes were identified in the draft genome of BinZNV2024. The transcription of these *rdhA* genes during the dechlorination of commercial V6, purified V6, and TCEP in culture ZNV was investigated using RT-qPCR (Fig. 4A through C; Fig. S7). It was observed that *rdhA12* and *rdhA9* were the only two genes with substantial transcription during the dechlorination of commercial V6, purified V6, and TCEP (Fig. 4A through C; Fig. S7), while the other 10 *rdhA* genes showed negligible or no expression (<0.01 transcripts per 16S rRNA gene). Notably, *rdhA12* consistently exhibited the highest transcript abundance across all three substrates: its levels reached 0.66, 0.51, and 1.1 transcripts per 16S rRNA gene during commercial V6, purified V6, and TCEP dechlorination, respectively. In contrast, *rdhA9* displayed significantly lower transcription under the same conditions, with levels of 0.54, 0.42, and 0.83 transcripts per 16S rRNA gene for commercial V6, purified V6, and TCEP (*P* < 0.05). This consistent disparity in transcript levels between *rdhA12* and *rdhA9* across all tested substrates suggests that *rdhA12* may play a more dominant role in the dechlorination processes mediated by culture ZNV, whereas *rdhA9* contributes to a lesser extent.

The expression of putative RDases during the dechlorination of commercial V6, purified V6, and TCEP in culture ZNV was assessed using metaproteomic analysis (Fig. 4D through F; Fig. S8). Among the 12 RdhAs detected, RdhA12 showed the highest expression levels, with normalized abundances of 2.26 × 10^10^, 2.16 × 10^10^, and 2.20 × 10^10^ in the presence of commercial V6, purified V6, and TCEP, respectively. RdhA9 exhibited the second-highest abundances, at 1.01 × 10^9^, 1.90 × 10^9^, and 1.46 × 10^9^ under the same conditions. The normalized abundances of the remaining putative RDases were all below 2.23 × 10^8^, which is two orders of magnitude lower than that of RdhA12, suggesting that the enzymes encoded by *rdhA12* and *rdhA9* are likely critical for the reductive transformation of commercial V6, purified V6, and TCEP. Furthermore, the consistently higher transcription and protein expression levels of *rdhA12* compared to *rdhA9* imply that *rdhA12* may play a more prominent role in the transformation of these compounds.

**Fig 5:**
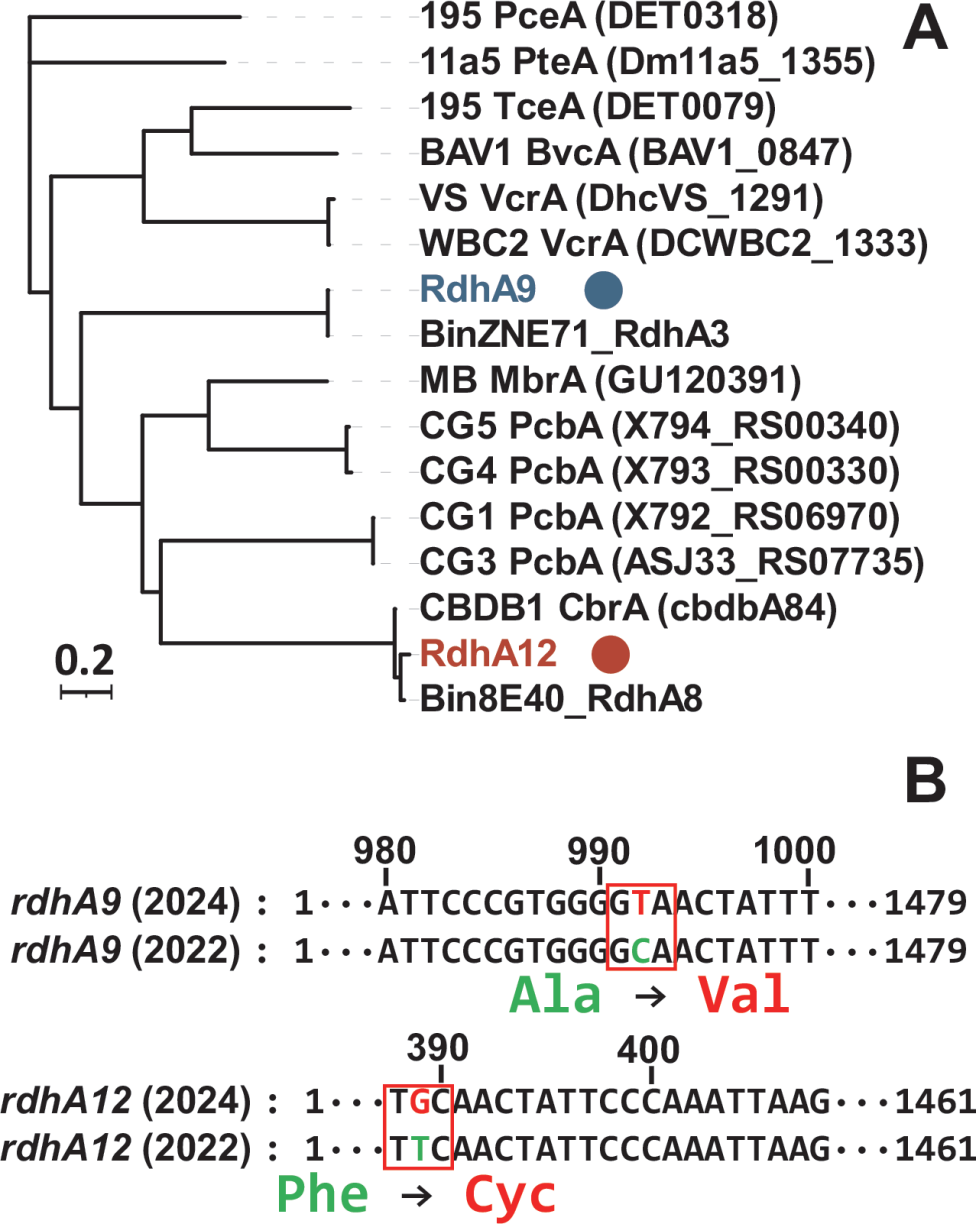
Phylogenetic analysis and non-synonymous substitutions of the *rdhA9* and *rdhA12* genes in the culture ZNV. (**A**) Phylogenetic tree comparing RdhA9 and RdhA12 sequences from ZNV with reference RDases from *D. mccartyi*. (**B**) Sequence divergence in *rdhA9* and *rdhA12* genes between metagenomes sequenced in 2022 and 2024.

Previous studies have demonstrated that multiple *rdhA* genes in *Dehalococcoides* can be transcribed and expressed during reductive dehalogenation (29–33). For example, multiple *rdhA* genes were highly transcribed during the growth of *D. mccartyi* 195 and *D. mccartyi* MB on perchloroethene or trichloroethene (28, 34). In addition, multiple RDases were detected in the proteomics of *D. mccartyi* 195 and CBDB1 grown with organohalide (22). Furthermore, four RDases were detected in *D. mccartyi* CBDB1 grown with the transformation of monobromobenzene to benzene (32). This suggests that, even with a single degradation pathway, *D. mccartyi* may express multiple RDases. Notably, RdhA12 exhibits 941% amino acid sequence identity with the putative RDase encoded by *Bin8E40_rdhA8*, which was previously detected in a draft genome of *D. mccartyi* derived from TCEP-dechlorinating culture 8E, originally enriched from e-waste polluted river sediments (Fig. S9) (16). *Bin8E40_rdhA8* also exhibited relatively high transcription levels during TCEP dechlorination, suggesting that the putative RDase encoded by *rdhA12* may serve as a key enzyme in the dechlorination of V6 and TCEP. Hug et al. established a classification framework for RDases, wherein sequences sharing ≥90% amino acid pairwise identity were categorized into ortholog groups (OGs) (35). Both *rdhA12* and *Bin8E40_rdhA8* encode putative RDases that cluster in OG53, a group that includes the characterized chlorobenzene reductive dehalogenase (CbrA) from the anaerobic organohalide-respiring bacterium *D. mccartyi* strain CBDB1 (Fig. 5A). However, *in vitro* dehalogenation assays of cells harvested from culture ZNV fed with commercial V6 suggested the putative RDase encoded by *rdhA12* could not transform 1,2,3,4-tetrachlorobenzene and 1,2,3-trichlorobenzene as CbrA did (data not shown). The distinct catalytic functions observed between RdhA12 and CbrA may be due to structural variations, potentially in key residues of their substrate-binding pockets or active-site architectures (36). The putative RDase encoded by *rdhA9* did not affiliate with the OGs in *D. mccartyi* Bin8E, and its specific role in the dechlorination of commercial V6, purified V6, and TCEP warrants further study.

Functional *rdhA* genes have the potential to serve as biomarkers for monitoring organohalide dechlorination in contaminated environments, thereby informing remediation strategies (37). The identification of potential Cl-OPEs *rdhA* genes in this study offers promising biomarkers for assessing the natural attenuation of commercial V6. These findings represent a crucial initial step toward evaluating, predicting, and monitoring the bioremediation of commercial V6 in contaminated sites.

### Nucleotide substitution in *rdhA* genes

Culture ZNV was maintained under selective pressure by serial transfers in medium containing elevated concentrations (160 mg L^−1^) of commercial V6 from July 2022 to May 2024 (Fig. S10). Comparative metagenomic sequencing was performed on DNA extracts from two time points: (i) culture ZNV exposed to 40 mg L^−1^ V6 (July 2022) and (ii) the adapted culture exposed to 160 mg L^−1^ V6 (May 2024). Through these analyses, we successfully assembled draft genomes of *D. mccartyi* strains BinZNV2022 and BinZNV2024, representing the microbial populations from the 2022 and 2024 sampling points, respectively.

The draft genomes of BinZNV2022 and BinZNV2024 each contained 12 *rdhA* genes, all of which are listed in Table S3. Among these, 10 out of the 12 *rdhA* genes were identical across the two draft genomes, suggesting a high degree of conservation of these genes originating from *D. mccartyi* in culture ZNV. However, both *rdhA9* and *rdhA12* exhibited a nucleotide substitution among the genomes (Fig. 5B). A nucleotide substitution at position 992 in *rdhA9*, when aligned with *rdhA9* (2022), led to a codon change from GCA (alanine) to GTA (valine). Similarly, a nucleotide substitution at position 389 in *rdhA12*, when aligned with *rdhA12* (2022), resulted in a codon change from TTC (phenylalanine) to TGC (cysteine). The occurrence of nucleotide substitution in functional *rdhA* genes has been reported previously (27, 38). However, whether these nucleotide substitutions affect the function of RdhA9 and RdhA12 remains unclear. Previous studies reported the consequences of nucleotide substitutions on the functional RDase. For instance, mutating a few residues within the active site of CfrA (a chloroform and trichloroethane-specific dehalogenase) can change its substrate preference to dichloroethane (39). This study provided no direct evidence linking nucleotide substitutions in the *rdhA* gene to functional changes between the original and adapted cultures, while the adapted culture, exposed to 160 mg L^−1^ of commercial V6 (four times higher than the 40 mg L^−1^ used for the original culture), maintained comparable dechlorination efficiency, demonstrating enhanced dechlorination capacity. We propose that nucleotide substitutions in the *rdhA* gene may confer dual advantages: resistance to high V6 concentrations and improved dechlorination performance. To ascertain whether prolonged exposure to elevated organohalide concentrations induces nucleotide substitutions in highly expressed *rdhA* genes and enhances dechlorination efficiency, further investigation is warranted.

### Environmental implications

Commercial V6, a mixture containing V6, TCEP, and impurities, poses significant environmental risks due to its persistence and potential carcinogenicity. This study provides the first experimental evidence for the effective dechlorination of both purified and commercial V6 formulations by a *D. mccartyi*-containing consortium. While reductive dehalogenases are known to mediate Cl-OPEs dechlorination, their functional dynamics in the dechlorination of complex mixtures remain unexplored. Using RT-qPCR and metaproteomic analyses, two *rdhA* genes (*rdhA9* and *rdhA12*) were co-transcribed, and their corresponding enzymes were expressed during exposure to commercial V6, purified V6, or TCEP. These results indicate that RdhA9 and RdhA12 may play an important role in the dechlorination of both TCEP and V6. These findings advance our understanding of key reductive dehalogenases involved in the reductive dechlorination of mixed Cl-OPEs. Future studies should focus on the interaction mechanisms between RdhA9 and RdhA12 during the dechlorination of mixed Cl-OPEs, which could be crucial for developing effective bioremediation technology for sites contaminated with Cl-OPEs. Furthermore, the potential involvement of other microorganisms, such as *Desulfobulbus* and *Desulfovibrio*, in the transformation of Cl-OPEs also warrants further investigation.

## MATERIALS AND METHODS

### Chemicals

Commercial V6 was purchased from MuseChem (Fairfield, NJ, USA). The authentic standard of V6 (95.8%) was purchased from Toronto Research Chemicals Inc. (Toronto, ON, Canada). TCEP (97%) and BCEP (97%) were purchased from Sigma-Aldrich (St. Louis, USA). Methanol and formic acid were of high-performance liquid chromatography-grade from Merck (Darmstadt, Germany) and CNW Technologies GmbH (Düsseldorf, Germany), respectively. Unless stated otherwise, other chemicals were purchased from MACKLIN Reagent Co., Ltd. (Shanghai, China).

### Purification of V6

A silica column was used to purify V6 from commercial V6. A total of 0.5 g of commercial V6 was dissolved in 500 µL of dichloromethane (DCM) and loaded onto the silica column. To elute TCEP, a 25% ethyl acetate in DCM (vol/vol) solution was applied until TCEP was no longer eluted. Subsequently, 50% ethyl acetate in DCM (vol/vol) was used to elute V6. The purified V6 had a quantified purity of 99.5% (wt/wt), and the concentration of TCEP in the purified V6 was below the detection limit (0.013 µM).

### Enrichment of *D. mccartyi*-containing mixed culture

Gutter sludge used for setting up microbial cultures was prepared as previously described (17). The background concentration of V6 in the gutter sludge was 287.8 ± 17.5 µg kg^−1^ dry weight. The enrichment of mixed cultures was conducted by adding 5.0 g of gutter sludge to 160 mL serum bottles containing 95 mL of defined medium with 2 mM titanium(III) nitrilotriacetic acid as a reducing agent (40, 41). The components of the defined medium were as described by Adrian et al. (40). The headspace was flushed with N_2_/CO_2_ (7:3, vol/vol). Commercial V6 (40.0 ± 3.7 mg L^−1^) was added in crystalline form directly to cultures. A mixture of short-chain organic acids (5 mM sodium acetate, 5 mM sodium lactate, 5 mM sodium propionate, and 0.1 mM sodium butyrate) was added as electron donors and carbon sources. Unless stated otherwise, all transfers were conducted about every 10 days with 5% vol/vol inoculum. After 20 successive transfers, the mixture of short-chain organic acids as carbon source and electron donor was replaced with hydrogen (12.4 mM) and acetate (10 mM). Additionally, ampicillin (1 g L^−1^) and 2-bromoethanesulfonic acid (2-BES, 1 mM) were added during transfers 20–40 to inhibit the growth of ampicillin-sensitive bacteria and methanogens. Following 40 successive transfers, this procedure resulted in the establishment of a methanogen-free V6-degrading mixed culture, designed as ZNV, in July 2022. Between July 2022 and May 2024, the culture was maintained with hydrogen (12.4 mM), acetate (10 mM), 2 mM titanium(III) nitrilotriacetic acid, and commercial V6 at a higher concentration of 160.0 ± 14.7 mg L^−1^. Transfers were conducted approximately every 10 days during this period. Unless stated otherwise, all subsequent experiments were conducted using Culture ZNV in 2024. The enrichment process of Culture ZNV is shown in Fig. S10.

### Transformation experiment setup

Transformation experiments were conducted in 60 mL serum bottles containing 25 mL of the defined medium mentioned above. Hydrogen (12.4 mM) and acetate (10 mM) were added as the electron donors and carbon sources, respectively. Ten microliters of V6 (600 g L^−1^), TCEP (300 g L^−1^), or commercial V6 (421 g L^−1^) dissolved in N,N-dimethylformamide (DMF) was added as electron acceptors. Control with sterilized cultures was conducted to monitor the abiotic loss (Fig. S3). Our preliminary study has shown that DMF was not utilized by the culture ZNV (data not shown). At each sampling time, bottles were sacrificed for chemical analysis and DNA/RNA extraction. Except for DNA, which was in duplicate for sequencing, all experiments were in triplicate. Five mL of liquid-phase sample was collected for measuring the concentrations of V6, TCEP, and transformation products; 30 µL of gas-phase sample was collected for measuring the concentrations of ethene; and 2 mL of each liquid-phase sample was collected for DNA and RNA extraction, which were subsequently used for qPCR and RT-qPCR, respectively. All bottles were incubated in an inverted position at 30°C in the dark without shaking.

### Instrumental analysis

V6, TCEP, and BCEP were quantified using Thermo Accela 1250 high-performance liquid chromatography coupled with a Thermo TSQ Vantage triple quadrupole MS. The details of the quantification are described in Text S1 and Table S4. Ethene in headspace was quantified using gas chromatography equipped with a flame ionization detector as described previously (17). Identification of impurities in commercial V6 and transformation products was conducted using a Thermo Ultimate 3000 UHPLC System equipped with an Orbitrap Fusion Lumos Tribrid Mass Spectrometer (Thermo Fisher Scientific, San Jose, CA) in +ESI mode. An XDB-C18 column (4.6 × 50 mm, 1.8 µm, Agilent, CA) was used for HPLC separation. The criteria for identification of Cl-OPEs transformation products were described previously (17). Briefly, the number of chlorine atoms on the Cl-OPEs was inferred from isotopic masses and ratios (Table S2 and Fig. S1). High-resolution m/z of [M+H]^+^ and [M+Na]^+^ were used to infer the formula, and MS/MS spectra were used to infer the structure (Fig. S3). DMF was quantified using an HPLC-UV system (LC-20AB, Shimazu, Kyoto, Japan) coupled with a reversed-phase ZORBAX Eclipse Plus C18 column (250 × 4.6 mm, 5 µm particle size; Agilent, Santa Clara, USA). The wavelength was set at 205 nm, and the mobile phase was methanol/water (vol/vol, 15/85) with a flow rate of 1 mL min^−1^ (42).

### DNA extraction, sequencing, and analyses, quantitative real-time PCR

Cells were harvested by centrifugation (15 min, 10,000 **×** *g* and 4°C). DNA was extracted using TIANamp Bacteria DNA Kit (TIANGEN Inc., Beijing, China) following the manufacturer’s protocol. Sequencing and analysis of metagenomes were conducted as previously described (17), but extracting draft genomes was performed using MetaWRAP (v1.2) with metaSPAdes (v3.13.0) for assembling and GTDB-Tk (v2.1.0) for assigning taxonomic classifications (43–45). Metagenomic sequencing was conducted using DNA extracted from culture ZNV in July 2022 and May 2024. And the draft genomes of *D. mccartyi* BinZNV2022 and BinZNV2024 were assembled, corresponding to the 2022 and 2024 metagenomic data sets, respectively. Additionally, the draft genome of *D. mccartyi* BinZNE was reassembled using the metagenomic sequences of culture ZNE reported previously (17). Sequencing of the 16S rRNA gene was also conducted as previously described, but analysis was performed using QIIME 2 following the Parkinson’s Mouse Tutorial (46). Maximum-likelihood phylogeny of *D. mccartyi* proteomes was built using PhyloPhlAn (v3.0.3) (47). The identification of *rdhA* genes was performed as previously described, and the maximum-likelihood phylogeny of RdhA amino acid sequence was built using RAxML (v8.2.12, -N 1000 -m PROTGAMMAILGX) (17, 48). The OGs of *rdhA* genes were identified using BLAST in the Reductive Dehalogenase Database (49). The qPCR enumeration of *D. mccartyi* was performed according to the method described previously (17). SGExcel FastSYBR Mixture (Sangon Biotech Co., Ltd., Shanghai, China) was used for the qPCR reaction.

### RNA extraction and RT-qPCR

Cells were harvested by centrifugation (15 min, 10,000 **×** *g* and 4°C) of 2 mL culture. RNA was extracted using TRIzol Universal Reagent (TIANGEN Inc., Beijing, China) following the manufacturer’s protocol. RNA was reverse transcribed into complementary DNA (cDNA) using the RevertAid RT Kit (Thermo Fisher Scientific Baltics UAB, Vilnius, Lithuania) following the manufacturer’s protocol. The qPCR reaction and thermocycling program were performed as previously described (17). The primers of *rdhA* genes designed with Primer Premier 6.0 are listed in Table 2. The specificities of primers were verified by melt curve analysis after amplification. The primer efficiencies were calculated with qPCR results of 1 to 10^−6^ gradient dilution DNA (Table 2). All the R^2^ of the DNA diluted gradient to C_t_ value plot were above 0.99, and the efficiencies ranged from 78% to 87%.

**TABLE 2:** Primers targeting *rdhA* genes used for RT-qPCR

Target	Primer name	Primer sequence (5' to 3')	Amp length (bp)	Efficiency (%)
*rdhA1*	ZNV_rdhA1F	TGGGAGGGTACTCCTGAAGAAA	70	83
	ZNV_rdhA1R	CCGTAGACGCTCCCATCAAC		
*rdhA2*	ZNV_rdhA2F	GCCGAAAAAGCCGAAACAT	71	83
	ZNV_rdhA2R	GCCGCGGTAATGGTTGAGT		
*rdhA3*	ZNV_rdhA3F	TTAGCCAGTGCCGGTATCG	73	87
	ZNV_rdhA3R	CAGAGACCACTTCATCAAGGTCAT		
*rdhA4*	ZNV_rdhA4F	GCAGGGTACTCCGGAGGAA	65	79
	ZNV_rdhA4R	GGGCTGCTCCGAAATGAA		
*rdhA5*	ZNV_rdhA5F	AGAACACTGCCGGATGTCATC	57	83
	ZNV_rdhA5R	CGGTTGGTCGTGCCGTAT		
*rdhA6*	ZNV_rdhA6F	TTCCTGCCAAAGCCAAATATATC	66	86
	ZNV_rdhA6R	GGCGTCTGGTGGATTCGTAA		
*rdhA7*	ZNV_rdhA7F	TTGGCTGGAGCAGGCATT	64	81
	ZNV_rdhA7R	CTTCGTCCAGGTCATGAAATACC		
*rdhA8*	ZNV_rdhA8F	CCAAAAAACCATGGTGGGTAAA	69	78
	ZNV_rdhA8R	GTTGCAAAAGGCTCAAATCCA		
*rdhA9*	ZNV_rdhA9F	TTGGATACGGACCCGGTTTA	71	83
	ZNV_rdhA9R	TGAGCATAAGGTACATCCAAAGCT		
*rdhA10*	ZNV_rdhA10F	CCGGCAAGCGTCTGTTCT	67	82
	ZNV_rdhA10R	GCACCAGCTACCGAGTTTGAA		
*rdhA11*	ZNV_rdhA11F	GTTATTGCCCCTACTCAAAGACTTG	68	84
	ZNV_rdhA11R	GAGCCGCGCCTGACTATCT		
*rdhA12*	ZNV_rdhA12F	CCCTTGGCATGGAGCATTAAT	63	84
	ZNV_rdhA12R	GGAGCGAGAGGCAAATCAGT		

To identify *rdhA* genes responsible for Cl-OPEs transformation, RT-qPCR assays without standard curves were conducted for each *rdhA* gene using cDNA as template (21, 30). Transcription of five *rdhA* genes was observed. The standard curves for each out of the five *rdhA* genes were constructed using dilution series (10-fold) of known concentrations of plasmid DNA, resulting in curves ranging from 10^2^ to 10^8^ gene copies per µL. The plasmid quantity was measured by UV spectrophotometry and provided as dried powder. The total amount of plasmid provided was 2–5 µg.

### Metaproteomic analysis

Cells harvested by centrifugation (15 min, 10,000 × *g* and 4°C) of 700 mL of cultures after 10 days of incubation were stored at −80°C. Proteins were extracted with 100 µL SDT buffer (4% SDS, 2% protease inhibitor [Roche, 04693132001-20 TABLETS], 50 mM Tris HCl, pH = 8.0), vortexed, and incubated at 95°C for 30 min. After samples were cooled to room temperature, an ultrasound was conducted with a cycle of 30 s working and 10 s stopping, with a total time of 20 min. Supernatant was stored at −80°C after centrifugation (10 min, 14,000 × *g* and 4°C). The proteins were quantified using the BCA Protein Assay Kit (TIANGEN Inc., Beijing, China). Proteins were digested and purified using QLBIO MagicOmics-MMB8X kit (Beijing Qinglian Biotech Co., Ltd., Beijing, China) following the manufacturer’s instructions. Peptide samples were dissolved in 10 µL of water with 0.1% formic acid and centrifuged (20 min, 14,000 × *g* and 4°C) before proteomics analyses using RIGOL L-3000 UHPLC system (RIGOL TECHNOLOGIES, INC.) coupled with Q Exactive HF-X Spectrometer (Thermo Fisher Scientific, Bremen, Germany) equipped with a Nanospray Flex (NSI) ion source. The separation was performed by a 25 cm-long column (100 µm inner diameter, packed using ReproSil-Pur C18-AQ 1.5-µm silica beads, Beijing Qinglian Biotech Co., Ltd., Beijing, China) at 60°C. The mobile phase consisted of methanol (A) and water (B, with 0.1% formic acid) at a flow rate of 300 nL min^−1^. The following elution gradient was used: 0–7 min 8%–12% A; 7–55 min 12%–30% A; 55–65 min 30%–40% A; 65–66 min 40%–95% A; 66–80 min 95% A. The parameters of MS1 were as follows: spray voltage, 3,000 V; ion transfer tube temp, 320°C; data-dependent acquisition mode with a full scan range, 350–1,500 m/z; resolution, 120,000 (200 m/z); AGC, 3 × 10^6^; maximum injection time for C-trap, 80 ms. The top 40 precursor ions based on intensity from the MS1 were selected for fragmentation using the high-energy collision dissociation method for MS2 detection. The parameters of MS2 were as follows: resolution of MS2, 15,000 (at 200 m/z), AGC of 5 × 10^4^; a maximum injection time, 45 ms; peptide fragmentation collision energy, 27%. All RAW files were analyzed using the Proteome Discoverer 3.1 suite (Thermo Fisher Scientific) with parameters: Sequest HT search engine, enzyme (trypsin), static modification (carbamidomethyl), dynamic modification (carbamidomethylation of cysteine residues, 57.02146 Da; oxidation of methionine residues, 15.99492 Da), precursor ion mass tolerance = 15 ppm, fragment ion mass tolerance = 0.02 Da, max missed cleavages = 2, minimum peptide length = 6. Percolator was used to filter peptide spectral matches and peptides to a false discovery rate (FDR) of less than 100%. After spectral assignment, peptides were assembled into 12 putative RDases annotated in this study and were further filtered based on the combined probabilities of their constituent peptides to a final FDR of 100%.

## Data Availability

The raw 16S rRNA gene amplicon sequences and metagenomic sequences have been deposited in the NCBI Sequence Read Archive under the accession number PRJNA912230 (SAMN46524870 and SAMN46541322).

## SUPPLEMENTAL MATERIAL

Supplemental material aem.01990-25-s0001.docx Text S1, Fig. S1 to S10, and Tables S1 to S4.

Graphical abstract aem.01990-25-s0002.eps Visual depiction of the study.

ASM does not own the copyrights to Supplemental Material that may be linked to, or accessed through, an article. The authors have granted ASM a non-exclusive, world-wide license to publish the Supplemental Material files. Please contact the corresponding author directly for reuse.
