# Supplementary material for: Dechlorination of mixed chlorinated organophosphate esters (V6 and TCEP) and associated reductive dehalogenase gene expression by Dehalococcoides mccartyi
Source: Appl Environ Microbiol. 2026 Jan 6;92(1):e01990-25. doi: 10.1128/aem.01990-25 (PMC12838431; doi:10.1128/aem.01990-25)
Supplement: Supplemental material — Text S1, Fig. S1 to S10, and Tables S1 to S4. [file aem.01990-25-s0001.docx]

**Supporting Information**

**Dechlorination of Mixed Chlorinated Organophosphate Esters (V6 and TCEP) and Associated Reductive Dehalogenase Gene Expression by *Dehalococcoides mccartyi***

Sen Yang^a,b,c,g^ Junhong Wu^a,b,c,g^, Qian Yang^a,b,c,g^, Yirong Deng^d^, Heli Wang^d^, Dan Li^e^, Lihua Yang^f^, Jianzhong Song^a,b,c^, Yin Zhong^a,b,c^*, Ping’an Peng^a,b,c^

**Summary: 26 pages, 1 text, 10 figures and 4 tables.**

^a^State Key Laboratory of Advanced Environmental Technology, Guangzhou Institute of Geochemistry, Chinese Academy of Sciences, Guangzhou 510640, China

^b^Guangdong Key Laboratory of Environmental Protection and Resources and Utilization, Guangzhou 510640, China

^c^Guangdong-Hong Kong-Maco Joint Laboratory for Environmental Pollution and Control Guangzhou 510640, China

^d^Guangdong Key Laboratory of Contaminated Sites Environmental Management and Remediation, Guangdong Provincial Academy of Environmental Science, Guangzhou 510045, China

^e^School of Environment and Civil Engineering, Dongguan University of Technology, Dongguan 523808, China

^f^South China Sea Resource Exploitation and Protection Collaborative Innovation Center, Guangdong Provincial Key Laboratory of Marine Resources and Coastal Engineering, School of Marine Sciences, Sun Yat-Sen University, Guangzhou 510275, China

^g^University of Chinese Academy of Sciences, Beijing 100049, China

*Corresponding author

Tel.: +86-20-85290142; Fax: +86-20-85290117

E-mail: [zhongyin@gig.ac.cn](mailto:zhongyin@gig.ac.cn)

**Text S1.** Quantification of V6, TCEP and BCEP.

**Fig. S1.** Isotopic patterns of Cl-OPEs and their transformation products in the culture ZNV.

**Fig. S2.** MS/MS spectra of Cl-OPEs and their transformation products in the culture ZNV.

**Fig. S3.** Control of the anaerobic transformation of commercial V6, purified V6 and TCEP, respectively.

**Fig. S4.** Proposed dechlorination pathways of impurities in commercial V6 by the culture ZNV.

**Fig. S5.** Growth of *Dehalococcoides* in the culture ZNV without addition of Cl-OPEs.

**Fig. S6.** Maximum-likelihood whole-genomic proteome phylogeny of *D. mccartyi*.

**Fig. S7.** RT-qPCR screening of all *rdhA* in culture ZNV.

**Fig. S8.** Number of peptides detected and coverage of seven putative RDases.

**Fig. S9.** Maximum-likelihood phylogenetic tree of RdhA from *D. mccartyi*.

**Fig. S10.** A schematic diagram showing enrichment process.

**Table S1.** Information on impurities in commercial V6 and their dechlorination products from UHPLC-Orbitrap Fusion TMS Spectra.

**Table S2.** Isotopic masses and ratios of Cl-OPEs and their dechlorination products from UHPLC-Orbitrap Fusion TMS Spectra.

**Table S3.** Nucleotide identity of *rdhA* genes in *D. mccartyi* BinZNV2024 and BinZNV2022.

**Table S4.** Quantitative and qualitative transitions of Cl-OPEs for Thermo TSQ Vantage triple quadrupole MS.

**Text S1.** Quantification of V6, TCEP and BCEP.

2,2-bis(chloromethyl)trimethylene bis(bis(2-chloroethyl) phosphate) (V6), tris(2-chloroethyl) phosphate (TCEP) and bis(2-chloroethyl) phosphate (BCEP) were quantified using Thermo Accela 1250 high-performance liquid chromatography coupled with a Thermo TSQ Vantage triple quadrupole MS. An XDB-C18 column (4.6 × 50 mm, 1.8 mm, Agilent, Ca) was used for HPLC separation. V6 was quantified using electrospray ionization positive mode (+ESI) with tris(1,3-dichloro-2-propyl) phosphate (TDCPP) as internal standard. The mass spectrometric parameters were as follows: spray voltage, 3000 V; vaporizer temperature, 350 °C; sheath gas pressure, 30 Arb; ion sweep gas pressure, 0 Arb; aux gas pressure, 35 Arb; capillary temperature, 270 °C; declustering voltage, 0 V; collision pressure, 1.8 mTorr. TCEP and BCEP were quantified as described previously (1). The mobile phase consisted of methanol (B) and water (B, with 0.1% formic acid) at a flow rate of 0.3 mL min^-1^. Quantitative and qualitative transitions of the chlorinated organophosphate esters (Cl-OPEs) are listed in Table S4.

**Fig. S1.** Isotopic patterns of Cl-OPEs and their transformation products in the culture ZNV. V6 (A); BCEP-BCMp-MCEP (B); BCEP-BCMHp (C); BCECpP (D); MCEP-BCMHp (E); BCEP-BCMp-P (F); CECpP (G); TCEP (H); BCEvP (I); BCEP (J); MCEP(K).

Experimental

Theoretical

Experimental

Theoretical

A

B

V6

BCEP-BCMp-MCEP

**Fig. S1.** Isotopic patterns of Cl-OPEs and their transformation products in the culture ZNV. V6 (A); BCEP-BCMp-MCEP (B); BCEP-BCMHp (C); BCECpP (D); MCEP-BCMHp (E); BCEP-BCMp-P (F); CECpP (G); TCEP (H); BCEvP (I); BCEP (J); MCEP(K). (Continued)

Experimental

Theoretical

C

BCEP-BCMHp

Experimental

Theoretical

D

BCECpP

**Fig. S1.** Isotopic patterns of Cl-OPEs and their transformation products in the culture ZNV. V6 (A); BCEP-BCMp-MCEP (B); BCEP-BCMHp (C); BCECpP (D); MCEP-BCMHp (E); BCEP-BCMp-P (F); CECpP (G); TCEP (H); BCEvP (I); BCEP (J); MCEP(K). (Continued)

Experimental

Theoretical

E

MCEP-BCMHp

Experimental

Theoretical

F

BCEP-BCMp-P

**Fig. S1.** Isotopic patterns of Cl-OPEs and their transformation products in the culture ZNV. V6 (A); BCEP-BCMp-MCEP (B); BCEP-BCMHp (C); BCECpP (D); MCEP-BCMHp (E); BCEP-BCMp-P (F); CECpP (G); TCEP (H); BCEvP (I); BCEP (J); MCEP(K). (Continued)

Experimental

Theoretical

G

CECpP

Experimental

Theoretical

H

TCEP

**Fig. S1.** Isotopic patterns of Cl-OPEs and their transformation products in the culture ZNV. V6 (A); BCEP-BCMp-MCEP (B); BCEP-BCMHp (C); BCECpP (D); MCEP-BCMHp (E); BCEP-BCMp-P (F); CECpP (G); TCEP (H); BCEvP (I); BCEP (J); MCEP(K). (Continued)

Experimental

Theoretical

I

BCEvP

Experimental

Theoretical

J

BCEP

**Fig. S1.** Isotopic patterns of Cl-OPEs and their transformation products in the culture ZNV. V6 (A); BCEP-BCMp-MCEP (B); BCEP-BCMHp (C); BCECpP (D); MCEP-BCMHp (E); BCEP-BCMp-P (F); CECpP (G); TCEP (H); BCEvP (I); BCEP (J); MCEP(K). (Continued)

Experimental

Theoretical

K

MCEP

**Fig. S2.** MS/MS spectra of Cl-OPEs and their transformation products in the culture ZNV. V6 (A); BCEP-BCMp-MCEP (B); BCEP-BCMHp (C); BCECpP (D); BCEP-BCMp-P (E); TCEP (F); BCEvP (G); BCEP (H).

A

B

V6

BCEP-BCMp-MCEP

**Fig. S2.** MS/MS spectra of Cl-OPEs and their transformation products in the culture ZNV. V6 (A); BCEP-BCMp-MCEP (B); BCEP-BCMHp (C); BCECpP (D); BCEP-BCMp-P (E); TCEP (F); BCEvP (G); BCEP (H). (Continued)

C

D

BCEP-BCMHp

BCECpP

**Fig. S2**. MS/MS spectra of Cl-OPEs and their transformation products in the culture ZNV. V6 (A); BCEP-BCMp-MCEP (B); BCEP-BCMHp (C); BCECpP (D); BCEP-BCMp-P (E); TCEP (F); BCEvP (G); BCEP (H). (Continued)

E

F

BCEP-BCMp-P

TCEP

**Fig. S2.** MS/MS spectra of Cl-OPEs and their transformation products in the culture ZNV. V6 (A); BCEP-BCMp-MCEP (B); BCEP-BCMHp (C); BCECpP (D); BCEP-BCMp-P (E); TCEP (F); BCEvP (G); BCEP (H). (Continued)

G

H

BCEvP

BCEP

**Fig. S3.** Control of the anaerobic transformation of commercial V6 (A), purified V6 (B) and TCEP (C), respectively. Control with sterilized cultures was conducted to monitor the abiotic loss.

**Fig. S4.** Proposed dechlorination pathways of impurities in commercial V6 by the culture ZNV.

**Fig. S5.** Growth of *Dehalococcoides* in the culture ZNV without addition of Cl-OPEs.

**Fig. S6.** Maximum-likelihood whole-genomic proteome phylogeny of *Dehalococcoides* *mccartyi* (*D. mccartyi*). Three subgroups (pinellas, victoria and cornell) of *D. mccarty* are displayed.

**Fig. S7.** RT-qPCR screening of all *rdhA* in culture ZNV. RT-qPCR were conducted with gene-specific primers (Table S4) to identify transcribed *rdhA* genes during dechlorination of commercial V6 (A), purified V6 (B) and TCEP (C), respectively. Figure A, B and C shared the same name of Y-axis title and legend.

**Fig. S8.** Number of peptides detected and coverage of seven putative RDases detected in proteomics of culture ZNV fed with commercial V6, purified V6 or TCEP.

**Fig. S9.** Maximum-likelihood phylogenetic tree of reductive dehalogenase (RdhA) from *D. mccartyi* BinZNV2024 obtained in this study, *D. mccartyi* Bin8E40 and BinZNE reported previously and other 26 representative *D. mccartyi* strain (i.e., 195, BTF08, GT, CBDB1, 11a5, NIT01, WBC-2, CG5, IBARAKI, JNA, DCMB5, BAV1, VS, GY50, CG3, CG1, MB, CG4, KBVC1, KBVC2, KBDCA1, KBDCA2, KBDCA3, KBTCE1, KBTCE2, KBTCE3). The RdhA selected from 26 representative *D. mccartyi* have been at least partially characterized and are indicated in black. The RdhA of *D. mccartyi* BinZNV2024, BinZNE and Bin8E40 are indicated in green, red and blue, respectively. Ortholog Groups (OGs) of RdhA are highlighted with OG numbers on the clade. Eight identified reductive dehalogenases (i.e., PceA, TceA, BvcA, VcrA, PteA, CrbA, MbrA and PcbA) are marked in the outside circle.

**Fig. S10.** A schematic diagram showing enrichment process. ^a^: 40 mg L^-1^ commercial V6; ^b^: 5 mM sodium acetate, 5 mM sodium lactate, 5 mM sodium propionate and 0.1 mM sodium butyrate; ^c^: 160 mg L^-1^ commercial V6; ^d^: 10 mM sodium acetate; ^e^: 12.4 mM hydrogen. All experiments were performed in triplicate, except for DNA samples, which were prepared in duplicate for sequencing.

**Table S1.** Information on impurities in commercial V6 and their dechlorination products from UHPLC-Orbitrap Fusion TMS Spectra.

| Abbreviation | RT (min) | Formula | [M + H]^+^ (m/z) | | Mass error (ppm) | RDB |
| --- | --- | --- | --- | --- | --- | --- |
|  |  |  | Theoretical | Experimental |  |  |
| BCEP-BCMHp | 9.67 | C_9_H_17_Cl_4_O_5_P | 376.9640 | 376.9644 | 0.882 | -0.5 |
| BCECpP | 9.03 | C_7_H_12_Cl_3_O_4_P | 296.9612 | 296.9612 | 0.032 | 0.5 |
| BCEvP | 8.23 | C_6_H_11_Cl_2_O_3_P | 232.9896 | 232.9895 | -0.398 | 0.5 |
| CECpP | 5.96 | C_5_H_9_Cl_2_O_4_P | 234.9688 | 234.9687 | -0.371 | 0.5 |

**Table S2.** Isotopic masses and ratios of Cl-OPEs from UHPLC-Orbitrap Fusion TMS Spectra. (Continued)

| Abbreviation | Retention time (min) | Experimental isotopic masses | Experimental isotopic ratio | Theoretical isotopic masses | Theoretical isotopic ratio | Number of Cl atoms |
| --- | --- | --- | --- | --- | --- | --- |
| V6 | 10.46 | 580.9151  582.9123  584.9094  586.9066  588.9038  590.9011  592.8986 | 51.78  100  82.21  35.64  8.27  1.13  0.12 | 580.9150  582.9121  584.9091  586.9062  588.9032  590.9003  592.8973 | 52.15  100  79.9  34.05  8.16  1.04  0.00 | 6 |
| BCEP-BCMp-MCEP | 9.89 | 518.9228  520.9200  522.9171  524.9139  526.9112  528.9090 | 63.77  100  65.52  20.91  3.37  0.21 | 518.9227  520.9198  522.9168  524.9139  526.9109  528.9080 | 62.58  100  63.92  20.43  3.26  0.21 | 5 |
| BCEP-BCMHp | 9.67 | 376.9643  378.9613  380.9583  382.9554  384.9524 | 80.37  100.00  50.14  10.79  1.00 | 376.9640  378.9611  380.9581  382.9552  384.9522 | 78.22  100  47.94  10.22  0.82 | 4 |
| BCECpP | 9.03 | 296.9612  298.9582  300.9553  302.9522 | 100  95.38  31.51  3.30 | 296.9612  298.9582  300.9553  302.9523 | 100  95.88  30.65  3.26 | 3 |
| TCEP | 8.92 | 284.9612  286.9582  288.9551  290.9520 | 100.00  97.19  33.94  3.88 | 284.9612  286.9582  288.9553  290.9523 | 100  95.88  30.65  3.26 | 3 |

**Table S2.** Isotopic masses and ratios of Cl-OPEs and their dechlorination products from UHPLC-Orbitrap Fusion TMS Spectra.

| Abbreviation | Retention time (min) | Experimental isotopic masses | Experimental isotopic ratio | Theoretical isotopic masses | Theoretical isotopic ratio | Number of Cl atoms |
| --- | --- | --- | --- | --- | --- | --- |
| BCEvP | 8.23 | 232.9895  234.9865  236.9836 | 100  65.49  10.23 | 232.9896  234.9866  236.9837 | 100.00  63.92  10.22 | 2 |
| MCEP-BCMHp | 8.11 | 314.9717  316.9690  318.9659  320.9635 | 100  87.13  27.94  5.35 | 314.9717  316.9688  318.9658  320.9629 | 100  95.88  30.65  3.26 | 3 |
| BCEP-BCMp-P | 7.23 | 456.9306  458.9276  460.9246  462.9220 | 76.32  100  45.61  9.61 | 456.9304  458.9274  460.9245  462.9215 | 78.22  100  47.94  10.22 | 4 |
| CECpP | 5.96 | 234.9687  236.9658  239.1438 | 100  53.72  7.59 | 234.9688  236.9659  238.9629 | 100  63.92  10.22 | 2 |
| BCEP | 5.28 | 222.9688  224.9658  226.9628 | 100.00  66.68  10.95 | 222.9688  224.9659  226.9629 | 100.00  63.92  10.22 | 2 |
| MCEP | 2.32 | 160.9765  162.9736 | 100.00  31.72 | 160.9765  162.9735 | 100.00  31.96 | 1 |

**Table S3.** Nucleotide identity of *rdhA* genes in *D. mccartyi* BinZNV2024 and BinZNV2022 obtained in 2024 and 2022, respectively.

| Name of *rdhA* genes | | Nucleotide identity (%) |
| --- | --- | --- |
| *D. mccartyi* BinZNV2024 | *D. mccartyi* BinZNV2022 |  |
| *rdhA1* (2024) | *rdhA1* (2022) | 100 |
| *rdhA2* (2024) | *rdhA2* (2022) | 100 |
| *rdhA3* (2024) | *rdhA3* (2022) | 100 |
| *rdhA4* (2024) | *rdhA4* (2022) | 100 |
| *rdhA5* (2024) | *rdhA5* (2022) | 100 |
| *rdhA6* (2024) | *rdhA6* (2022) | 100 |
| *rdhA7* (2024) | *rdhA7* (2022) | 100 |
| *rdhA8* (2024) | *rdhA8* (2022) | 100 |
| *rdhA9* (2024) | *rdhA9* (2022) | 99.93 |
| *rdhA10* (2024) | *rdhA10* (2022) | 100 |
| *rdhA11* (2024) | *rdhA11* (2022) | 100 |
| *rdhA12* (2024) | *rdhA12* (2022) | 99.93 |

**Table S4.** Quantitative and qualitative transitions of Cl-OPEs for Thermo TSQ Vantage triple quadrupole MS.

| Mode | Compound | Quantitative transition | | | | Qualitative transition | | | |
| --- | --- | --- | --- | --- | --- | --- | --- | --- | --- |
|  |  | Parent Mass (m/z) | Product Mass (m/z) | Collision E | S-Lens | Parent Mass (m/z) | Product Mass (m/z) | Collision E | S-Lens |
| +ESI | V6 | 582.90 | 98.98 | 46 | 113 | 582.90 | 360.80 | 16 | 113 |
|  | TCEP | 284.90 | 98.98 | 24 | 72 | 284.90 | 160.97 | 24 | 72 |
|  | TDCPP | 430.90 | 98.98 | 20 | 113 | 430.90 | 208.90 | 16 | 113 |
|  | TCPP | 327.01 | 98.98 | 29 | 61 | 327.01 | 194.98 | 10 | 61 |
| -ESI | BCEP | 221.1 | 35.00 | 14 | 46 | 223.1 | 37.00 | 14 | 46 |
|  | BCPP | 249.00 | 35.00 | 12 | 38 | 251 | 37.00 | 12 | 38 |

**REFERENCES**

(1) Yang, S.; Wu, J.; Wang, H.; Yang, Q.; Zhang, H.; Yang, L.; Li, D.; Deng, Y.; Zhong, Y.; Peng, P. a. New dechlorination products and mechanisms of tris(2-chloroethyl) phosphate by an anaerobic enrichment culture from a vehicle dismantling site. *Environ. Pollut.* **2023**, 122704.
